# Structure and processes of emergency observation units with a geriatric focus: a scoping review

**DOI:** 10.1186/s12877-021-02029-9

**Published:** 2021-02-01

**Authors:** Pieter Heeren, Annabelle Hendrikx, Janne Ceyssens, Els Devriendt, Mieke Deschodt, Didier Desruelles, Johan Flamaing, Marc Sabbe, Koen Milisen

**Affiliations:** 1 Department of Public Health and Primary Care, Academic Centre for Nursing and Midwifery, KU Leuven, Kapucijnenvoer 35/4, 3000 Leuven, Belgium; 2 Department of Geriatric Medicine, University Hospitals Leuven, Herestraat 49, 3000 Leuven, Belgium; 3 Research Foundation Flanders, Egmontstraat 5, 1000 Brussels, Belgium; 4 Department of Public Health and Primary Care, Gerontology and Geriatrics, KU Leuven, Herestraat 49, 3000 Leuven, Belgium; 5 Department of Public Health, Nursing Science, University of Basel, Bernoullistrasse 28, 4056 Basel, Switzerland; 6 Faculty of Medicine and Life Sciences, Healthcare and Ethics, UHasselt, Martelarenlaan 42, 3500 Hasselt, Belgium; 7 Department of Emergency Medicine, University Hospitals Leuven, Herestraat 49, 3000 Leuven, Belgium; 8 Department of Public Health and Primary Care, Emergency Medicine, KU Leuven, Kapucijnenvoer 35/4, 3000 Leuven, Belgium

**Keywords:** Acute care, Emergency department, Observation unit, Older adults, Geriatric emergency medicine, Comprehensive geriatric assessment

## Abstract

**Background:**

Combining observation principles and geriatric care concepts is considered a promising strategy for risk-stratification of older patients with emergency care needs. We aimed to map the structure and processes of emergency observation units (EOUs) with a geriatric focus and explore to what extent the comprehensive geriatric assessment (CGA) approach was implemented in EOUs.

**Methods:**

The revised scoping methodology framework of Arksey and O’Malley was applied. Manuscripts reporting on dedicated areas within hospitals for observation of older patients with emergency care needs were eligible for inclusion. Electronic database searches were performed in MEDLINE, EMBASE and CINAHL in combination with backward snowballing. Two researchers conducted data charting independently. Data-charting forms were developed and iteratively refined. Data inconsistencies were judged by a third researcher or discussed in the research team. Quality assessment was conducted with the Methodological Index for Non-Randomized Studies.

**Results:**

Sixteen quantitative studies were included reporting on fifteen EOUs in seven countries across three continents. These units were located in the ED, immediately next to the ED or remote from the ED (i.e. hospital-based). All studies reported that staffing consisted of at least three healthcare professions. Observation duration varied between 4 and 72 h. Most studies focused on medical and functional assessment. Four studies reported to assess a patients’ medical, functional, cognitive and social capabilities. If deemed necessary, post-discharge follow-up (e.g. community/primary care services and/or outpatient clinics) was provided in eleven studies.

**Conclusion:**

This scoping review documented that the structure and processes of EOUs with a geriatric focus are very heterogeneous and rarely cover all elements of CGA. Further research is necessary to determine how complex care principles of ‘observation medicine’ and ‘CGA’ can ideally be merged and successfully implemented in clinical care.

**Supplementary Information:**

The online version contains supplementary material available at 10.1186/s12877-021-02029-9.

## Background

Between 12 and 24% of patients presenting to emergency departments (ED) are 65 years or older [1]. This growing segment of the ED population includes a vulnerable subgroup, which is characterized by multimorbidity, polypharmacy and reduced physical and psychosocial reserves. Under these circumstances, older ED patients are at increased risk for unfavorable outcomes, such as death, prolonged ED length of stay (LOS), unnecessary admission and unplanned readmission, compared to their younger counterparts [2–5]. To enhance these outcomes and better meet the complex needs of this vulnerable group, geriatric emergency guidelines recommend to integrate principles of comprehensive geriatric assessment (CGA) in emergency care [6, 7]. CGA has been defined as “a multidimensional, interdisciplinary diagnostic process focusing on determining a vulnerable older person’s medical, functional, cognitive and social capabilities in order to develop a coordinated and integrated plan for treatment and long term follow up” [8].

As CGA can be time consuming and EDs can have short targets for LOS (e.g. four hour rule in United Kingdom and Australia [9, 10]), integrating geriatric emergency guidelines in the regular ED setting is perceived challenging. Indeed, integration of these guidelines seems more compatible with the concept of emergency observation units (EOUs) [11–13]. These units traditionally focus on patients requiring a longer period of time (often 8–24 h) for further diagnostic testing, reassessment, therapeutic interventions or consultations, which is beyond the scope of the conventional ED stay. Generally, EOUs do not qualify for “buffering” patients in need of an inpatient bed [11, 14]. The reported benefits of EOUs for general patient populations at the patient, hospital and care system level include higher patient satisfaction, shorter LOS, decreased ED crowding, fewer inpatient admissions, and lower cost [15–17]. However, the certainty of the reported evidence is very low [18]. For vulnerable older adults, the additional available time in EOUs provides an opportunity for comprehensive, interdisciplinary assessment and focused geriatric care as a means for more appropriate risk stratification, management or disposition planning [11, 12].

As we could not identify any published review on EOUs with a geriatric focus, a scoping review was conducted to map and summarize the existing literature on this topic. Our aim was to explore the structure and processes of EOUs with a geriatric focus in an international context. More specifically, we explored to what extent the geriatric focus in EOUs corresponded to the concept of CGA, which is considered the gold standard approach in geriatric care models [8, 19].

## Methods

A scoping review was conducted, using the refined methodological framework of Arskey and O’Mally [20, 21]. This manuscript was reported using the PRISMA guidelines and its extensions for Scoping Reviews [22].

### Identification of relevant studies

Two phases were used to identify relevant studies. First, electronic database searches were conducted after tailoring the search strategy to the thesaurus of MEDLINE, EMBASE and CINAHL. Final search strings are available in supplementary Table S1. These comprised three concepts (i.e. emergency medical services AND older patients AND observation units) and had one restriction: only papers published in English, Dutch or French were considered for inclusion. Second, reference lists of pertinent literature review studies were screened to find additional relevant publications (i.e. backward snowballing).

### Selection of studies

A four-stage study selection process was conducted. First, duplicates were removed with Endnote software. Second, all records were screened for suitability based on title and abstract. In this stage, the three concepts of the final search strings were used as initial selection criteria. PH screened all identified records, while JC and AH each screened half. Third, each study, considered potentially relevant by at least one researcher in the previous stage, underwent full-text screening. This was conducted by PH, JC and AH, who completed this independent of each other. During this stage, iterative consensus meetings were organised to discuss how initial selection criteria could be refined, taking into account the retrieved manuscripts and the study aim. Fourth, the reference list of included studies was screened to find additional relevant publications (i.e. backward snowballing).

The final inclusion criteria set out four requirements for including a paper. The first three delineated the population (i.e. adults of 65 years and older or a median sample age of at least 70 years old), setting (i.e. dedicated areas within hospitals for observation of patients during a predefined time period following emergency admission) and design (i.e. quantitative and qualitative studies reporting primary data analyses). The fourth inclusion criterion was having a geriatric focus. This was defined as “providing some form of additional assessment or intervention for older adults compared to usual care from the perspective that older adults have different needs than younger patients”. Studies reporting on pathology specific interventions (e.g. delirium, hip fracture) were excluded, as well as care models on inpatient wards or intensive care units. Other exclusion criteria focused on study design (i.e. review papers, editorials, letters to the editor, published abstracts and conference proceedings) and the extensiveness of reporting. The latter implied exclusion of manuscripts that did not describe intervention components, processes or outcome measures.

### Data charting

The initial data charting forms were based on two items: Conley and colleagues’ overview of key elements to consider when establishing an observation unit [23] and Moseley and colleagues’ summary of observation unit characteristics [11]. Initial data-charting forms included methodological items (i.e. study characteristics and quality appraisal items) and general characteristics of EOUs (e.g. design, staffing, admission policy, workflow). An iterative approach (i.e. continually updating the data-charting forms) was used by three researchers (PH, JC and AH) to elaborate these characteristics based on included studies. Consensus meetings within the research team guided refinement of data charting.

The methodological quality of quantitative studies was described with the twelve-item Methodological index for non-randomized studies (MINORS) [24]. Each item was assigned a score zero (i.e. not reported), one (i.e. reported but inadequate) or two (i.e. reported and adequate). Included studies were assessed independently by PH (who scored all studies) and JC or AH (who each scored half of the studies). MS assessed inconsistent scores together with PH, JC and AH. The Standard for Reporting Qualitative Research was selected to assess the quality of qualitative studies [25].

### Sorting, summarizing and reporting results

Data were grouped by methodological and EOU-specific characteristics of each included paper. EOU-specific data were initially mapped according structural and procedural characteristics of EOUs and subsequently discussed according the key elements of the CGA definition [8] (i.e. interdisciplinary processes, target population, multidimensionality and plan for treatment and follow-up).

## Results

### Identification and selection of relevant studies

**Fig. 1:**
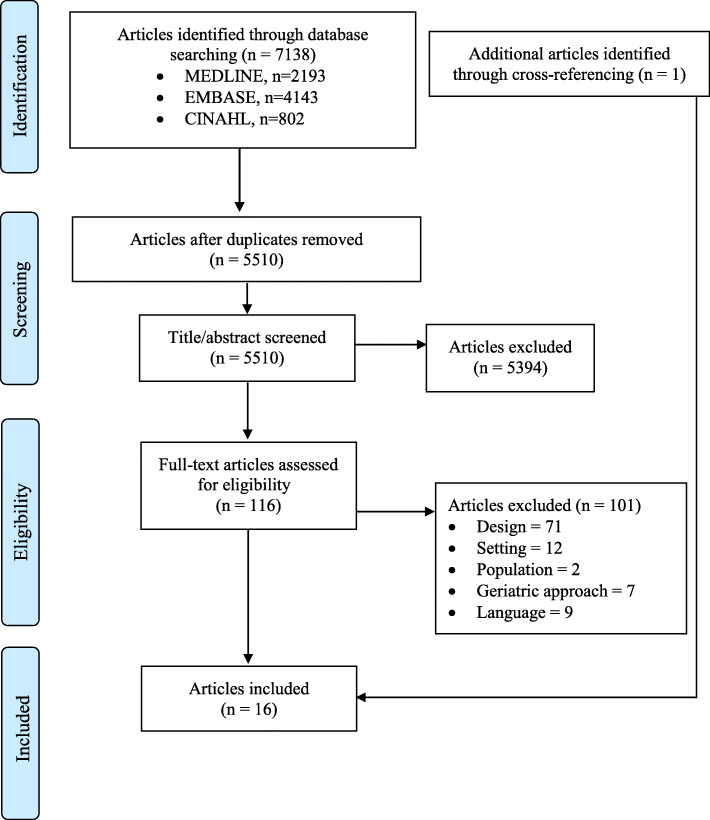
PRISMA flowchart

Database searches resulted in 7138 papers. After removing duplicates (*n* = 1628), 5510 papers remained. After screening of titles and abstracts 5394 papers were excluded. Full-text screening was conducted for 116 papers, resulting in 15 included studies. We included one additional study through screening the reference lists of the included studies. Figure 1 shows the flowchart of the study identification and selection process.

### Characteristics of included studies (Table 1)

The sixteen included studies reported on fifteen different EOUs with a geriatric focus in seven countries: six in the UK [26–31], four in Denmark [32–35], two in Australia [36, 37] and one in Singapore [38], Hong Kong [39], Switzerland [40] and the USA [41]. All publications had a quantitative design. Six papers reported retrospective data collection [29, 31, 36, 37, 39, 41]. Papers with prospective data collection used following designs: observational study (*n* = 3) [26, 29, 40], pre-post study (*n* = 2) [26, 27], system redesign study [30], non-randomized quasi-experimental trial [33], two-way factorial randomized clinical trial [32], pragmatic randomized clinical trial [35] and randomized controlled trial [28]. The only multicenter study collected data in two hospitals [28]. Risk for bias of included studies varied from moderate to high (supplementary Table S2; e.g. seven studies included consecutive patients, baseline equivalence of groups was considered adequate in two studies).

**Table 1:** Study characteristics

Study	Country	Study design	Population	Sample	Age^a^	Care model name
Anpalahan 2002 [36]	Australia	Retrospective, record review study; monocentre	≥ 70 years General medical patients	*n* = 500	NR	Rapid assessment medical unit
Bruun 2018 [32]	Denmark	Prospective, two-way factorial randomised clinical trial; monocentre	≥ 65 years Non-trauma patients at risk of functional decline	Group I; *n* = 82 Group II; *n* = 84 Group III; *n* = 86 Group IV; *n* = 84	78 (72–85)	SSU
Chu 2007 [26]	UK	Prospective, observational study; monocentre	≥ 60 years	*n* = 120	77 (60–96)	Short-stay medical unit
Conroy 2014 [27]	UK	Prospective, pre-post study (historical cohort); monocentre	≥ 85 years	*n* = 6895 (CG) *n* = 9035 (IG)	NR	Emergency Frailty Unit
Edmans 2013 [28]	UK	Prospective, randomised controlled trial; multicentre (2 locations)	≥ 70 years Length of stay ≤72 h ISAR score ≥ 2/6	*n* = 217 (CG) *n* = 216 (IG)	83 (±7)	Acute Medical Assessment Unit
Foo 2012 [38]	Singapore	Prospective, pre-post study; monocentre	≥ 65 years Community-dwelling No poor premorbid cognition or functionality	*n* = 172 (CG) *n* = 315 (IG)	75 (NR) in CG 76 (NR) in IG	Emergency Department Observation Unit
Khan 1997 [29]	UK	Retrospective, observational study; monocentre	≥ 65 years	*n* = 502	NR	Short-stay ward
Leung 2019 [39]	Hong Kong	Retrospective, parallel group study; monocentre	≥ 65 years Living alone	*n* = 40 (CG) *n* = 150 (IG)	82.1 (±8.2) in CG 83.5 (±7.7) in IG	Frailty unit
Misch 2014 [40]	Switzerland	Prospective, observational delayed type cross-sectional diagnostic study; monocentre	Non-trauma patients emergency severity index score 2 or 3 non-specific complaints	*n* = 669	81 (72–87)	Emergency Department Observation Unit
Nielsen 2018 [33]	Denmark	Prospective, non-randomised quasi-experimental trial; monocentre	≥ 65 years Non-trauma Community-dwelling	*n* = 231 (CG) *n* = 144 (IG)	78 (±9) in CG 81 (±8) in IG	SSU
Ong 2012 [37]	Australia	Retrospective, case-control study; monocentre	≥ 65 years 4 most common diagnosis-related groups	*n* = 42 (CG) *n* = 47 (IG)	80 (±8) in CG 84 (±8) in IG	Medical Assessment Unit
Silvester 2012 [30]	UK	Prospective system redesign study; monocentre	≥ 75 years	*n* = 16,953	NR	Frailty unit
Southerland 2018 [41]	USA	Retrospective, chart review study; monocentre	≥ 65 years	*n* = 221	73 (±7)	Emergency Department Observation Unit
Strøm 2017 [34]	Denmark	Prospective, observational study; monocentre	≥ 75 years Non-emergent triage score internal medicine disease	*n* = 225 (SSU) *n* = 225 (IMW)	82 (78–86) in SSU 82 (78–86) in IMW	SSU
Strøm 2018 [35]	Denmark	Prospective, pragmatic randomised clinical trial; monocentre	≥ 75 years Less urgent triage score internal medicine disease	*n* = 208 (SSU) *n* = 210 (IMW)	81 (77–86) in SSU 82 (78–86) in IMW	SSU
Taylor 2016 [31]	UK	Retrospective, pre-post study; monocentre	> 75 years Medical patients	*n* = 398 (CG) *n* = 413 (IG)	85 (75–101) in CG 84 (75–101) in IG	Comprehensive Older Person’s Evaluation Zone

*CG* Control group, *IG* Intervention group, *IMW* Internal medicine ward, *NR* Not reported, *ISAR* Identification of Senior At Risk, *SSU* Short-stay unit, *UK* United Kingdom, *USA* United States of America

^a^median (range) or mean (±standard deviation) in years

### Structure of EOUs with a geriatric focus

#### Unit design (supplementary Table S3)

Thirteen papers reported the location of the EOU. These had been positioned at three places: in the ED [32, 33, 39–41], immediately next to the ED [27, 29, 34, 35, 38] and remote from the ED (i.e. hospital-based) [26, 30, 37]. The available bed count varied from 6 to 32 beds and varied according to demand [26, 27, 29, 31, 34, 35, 37–39, 41]. One Danish EOU had six chairs available for daytime patients [34, 35]. Four studies reported a distinct zone specifically reserved for older patients [27, 30, 31, 39].

#### Staffing (supplementary Table S4)

Fifteen out of sixteen studies reported that staffing consisted of at least three healthcare professions [26–39, 41]. In thirteen studies, the interdisciplinary team comprised at least one physician, nurses and one or more allied health care professionals [26–28, 30–33, 35–39, 41]. These included physiotherapists (*n* = 14), occupational therapists (*n* = 12), social workers (*n* = 6), pharmacists (*n* = 3) and/or discharge planning coordinators (*n* = 1). Extended nursing roles included mental health liaison nurse (*n* = 1) [31], nurse case manager (*n* = 4) [27, 31, 39, 41] and advanced nurse practitioner/advanced practice provider (*n* = 3) [36, 38, 41]. Two studies did not report the presence of a nurse [29, 34]. Input of a geriatrician was reported in six studies and varied between a consultant role and complete coverage during daytime [27, 28, 30, 31, 39, 41].

Seven studies reported some details on availability of the interdisciplinary team. Three and two studies reported operating periods from Monday until Friday [29, 31, 33] and from Monday until Saturday [38, 41], respectively. Two studies reported daily geriatrician coverage [27, 30].

### Processes of EOUs with a geriatric focus

#### Admission policy (supplementary Table S5)

Seven publications reported whether the admission procedure of the EOU was ‘closed’ (i.e. admission only after assessment by ED physician; *n* = 5) [26, 29, 34, 35, 41] or ‘open’ (i.e. admission after referral of a physician, such as a general practitioner; ED evaluation may or may not be required; *n* = 2) [31, 37].

Fourteen studies described that the EOU focused on subacute patients with potential for discharge within a predefined observation period, which varied between 4 and 72 h. Five, four and two papers reported a targeted observation period of 72 h [26, 28, 34, 35, 39], 24 h [27, 29, 40, 41] and 48 h [33, 36], respectively. Three studies reported flexible observation periods, ranging between 4 and 24 h [38], 36–48 h [37] and 48–72 h [32].

One study used an international validated screening tool (i.e. Identification of Seniors at Risk or ISAR) to guide selection of older patients for a geriatric approach [28]. Another study reported that all older patients being identified with at least one of four predefined criteria (i.e. falls, delirium, dementia or care home/intermediate care residents) were eligible for CGA [31]. Additional criteria that were used for narrowing down the observation population focused on pathology (e.g., only patients suffering from specific conditions [38] or fulfilling criteria of chief complaint-focused protocols [41]), social profile (e.g., only community-dwelling patients [38, 39]) and premorbid cognition or function (e.g., no patients with advanced dementia or bed-bound profiles [38, 39]). One study reported no details on admission criteria [30].

#### Procedural elements of EOUs with a focus on older patients (Table 2)

To manage patients within the predefined observation period, all studies except one reported to use fast-track principles (*n* = 15) [26, 27, 29–41]. These comprised care pathways to streamline patients from the ED into the observation unit (*n* = 2) [27, 31], early senior medical input (*n* = 6) (e.g. geriatricians of a frailty unit could have an in-reach function to the ED) [26, 27, 30, 31, 37, 40] and fast-track access to diagnostic tests and therapeutic procedures (*n* = 8) [26, 31, 34, 35, 37, 39–41]. Other fast-track principles comprised early initiation of discharge planning (*n* = 11) [26, 29–31, 35–41] and stimulation of self-care or early mobilization (*n* = 2) [32, 35].

**Table 2:** Procedural elements of observation stays with geriatric focus

	Anpalahan 2002 [36]	Bruun 2018 [32]	Chu 2007 [26]	Conroy 2014 [27]	Edmans 2013 [28]	Foo 2012 [38]	Khan 1997 [29]	Leung 2019 [39]	Misch 2014 [40]	Nielsen 2018 [33]	Ong 2012 [37]	Silvester 2012 [30]	Southerland 2018 [41]	Strøm 2017 [34]	Strøm 2018 [35]	Taylor 2016 [31]
**Fast-track principles**																
Diagnostic tests/treatment			X					X	X		X		X	X	X	X
Early senior medical input			X	X					X		X	X				X
Stimulation of self-care / early mobilization		X								X					X	
Referral pathway to observation unit				X												X
Early initiation of discharge planning	X		X			X	X	X	X		X	X	X		X	X
**(Early) Geriatric-focused assessment**																
Medical				X	X	X		X	X			X	X			X
Functional		X		X	X	X		X	X	X	X	X	X	X	X	X
Cognitive	X			X	X	X										X
Psychological				X	X	X										X
Social				X	X	X		X			X	X	X			X
Drug review				X	X								X			X
Unspecified			X		X		X		X							
All four items of CGA				X	X	X										X
**Unit rounds**	X			X			X	X								
**Interdisciplinary collaboration**																
Interdisciplinary coordination	X			X	X	X		X	X					X	X	X
Team meeting				X		X			X		X					X
Case discussion				X		X										
**Observation pathway**																
Frailty pathway								X					X			
ED-based fragility fracture pathway													X			
**Follow-up**																
Post-discharge follow-up		X		X	X	X	X	X	X	X		X	X			X
Transmural information transfer		X		X	X	X	X			X						
Transmural pathways				X	X					X						X

*CGA* Comprehensive geriatric assessment, *ED* Emergency department

Interdisciplinary processes included making proactive and integrated referrals to available consultants and/or ancillary services (e.g. social work, physical therapy, occupational therapy) as part of standard observation care [26–39, 41]. Reported initiatives to improve standard care were very heterogeneous. One study reported integrating systematic cognitive screening in routine assessment by nurses or junior physicians [36]. Two studies described an initiative for systematic functional assessment and early rehabilitation conducted by physiotherapists or occupational therapists [32, 33]. One study integrated geriatric assessment by emergency nurses trained in geriatric care [38]. Other initiatives comprised the introduction of specific geriatric protocols (i.e. frailty protocol and fragility fracture protocol) (*n* = 2) [39, 41] or the integration of geriatrician-led CGA (*n* = 3) [27, 28, 31]. Regarding comprehensiveness of assessments, five studies clearly reported assessing cognitive function [27, 28, 31, 36, 38]. In total, four of the included studies reported to assess a patient’s medical, functional, cognitive and social capabilities [27, 28, 31, 38].

Nine studies reported who coordinated the interdisciplinary team. Seven studies had a physician-led interdisciplinary process (i.e. emergency physician, acute physician or geriatrician) [27, 28, 31, 34, 35, 39, 40]. In one study, advanced nurse practitioners were available to work across disciplines and coordinate patient management [36]. Another study described that ED nurses reported geriatric assessment findings to an ED physician or a geriatric nurse clinician [38]. Use of case discussion and team meetings were reported in two [27, 38] and five [27, 31, 37, 38, 40] studies, respectively. Reported frequencies of team meetings were once daily [37], twice daily [31] and twice weekly [38].

Eleven studies described reporting some form of post-discharge follow-up [27–33, 38–41]. Its extensiveness was variable, ranging between one specific option (e.g. immediate rehabilitation or not) and a package of follow-up possibilities in primary (i.e. general practitioner), secondary (e.g. geriatric outpatient clinics), community (e.g. home nursing), intermediate (e.g. rehabilitation hospital) and/or social care [27, 28, 32, 33, 38]. Four studies described these initiatives as ‘transmural or direct referral pathways’. [27, 28, 31, 33] Six of the studies also reported to engage in transmural information transfer [27–29, 32, 33, 38].

## Discussion

Although the conceptual integration of EOUs and CGA seems highly compatible, only four studies [27, 28, 31, 38] described a geriatric focus meeting all main elements of the CGA definition [8].

### Interdisciplinary processes

The low amount of CGA-labelled studies could not be attributed to a lack of interdisciplinary processes (i.e. availability of at least two disciplines collaborating and sharing expertise to deliver optimal care [8]), as all included studies met this CGA element. Even more, all studies, except for one, reported availability of at least three disciplines, with physicians, nurses, physical therapists and occupational therapists as most frequent reported members. Remarkably, only seven studies reported availability of at least one geriatric practitioner (e.g. geriatrician or nurse with geriatric expertise) [27, 28, 30, 31, 38, 39, 41] Absence of a geriatric practitioner in the current review can be explained by three reasons. First, staffing characteristics of routine ED care and interventions (e.g. minimal educational backgrounds, fulltime equivalent availability, roles and responsibilities of different interdisciplinary team members) were often poorly described or not reported. Second, in an international perspective, shortage of geriatricians and nurses with geriatric expertise remains a problem [42–44]. Third, specific for the ED and EOU setting, absence of geriatric practitioners can be caused by the limited ability to bill or charge for geriatric interventions [42]. One might say, with or without a dedicated geriatric practitioner, an EOU should always strive delivering the most appropriate care for older patients. Clearly, in absence of a geriatric practitioner, the individual role of all interdisciplinary team members and their mutual collaboration becomes more essential [42].

## Target population

Admission criteria varied widely from one setting to another but appeared appropriate for local feasibility, as no study reported challenges with implementing. Clinicians contemplating to initiate geriatric-focused observation services, need to consider both geriatric and observation selection criteria. Regarding geriatric selection criteria, it is remarkable that only one study reported usage of an international validated geriatric screening tool, which continues to be promoted as best practice despite its limitations [28, 45–47]. The value of other geriatric selection criteria of included studies remains unknown, as their description was often insufficiently detailed or relied on clinical judgement only. For example, Taylor and colleagues defined a set of four objective and straightforward criteria to guide patient selection (i.e. falls, delirium, dementia or care home/intermediate care), but no information was reported on how these concepts were operationalized (e.g. use of validated screening tools/definitions, screening moment, person performing the screening) [31].

Observation selection criteria of included studies focused predominantly at avoiding unnecessary admissions. This means that all patients requiring a prolonged ED stay without clear qualification for inpatient care were referred to the observation unit if possible (e.g. social problems). As the general accepted ‘discharge to home’ and ‘inpatient conversion’ rates are 80 and 20%, respectively, it is clear that ‘observational failure’ (i.e. admission of an observation patient) is a part of observation care, as well [23]. For older patients, this means that EOUs can be an ideal area to exclude atypical presentation of severe pathology in patients with non-specific complaints [3, 40].

## Multidimensionality

The multidimensional character of assessments, described in the included studies, is very questionable and should get more attention, as only four studies clearly reported to assess a patient’s medical, functional, cognitive and social capabilities [27, 28, 31, 38]. However, one might consider that these aspects were poorly reported, as well. Therefore, authors, reviewers and editors should make more efforts to ensure that readers of a manuscript can clearly understand the content of an assessment and by extension the entire intervention if applicable. The TIDieR guidelines can be helpful for this purpose [48]. Important to know for non-geriatric trained caregivers in EOUs is that subjective, self-reported patient or caregiver data might be unreliable. Therefore a (C) GA uses objectively, validated instruments to assess the risk for specific problems [49, 50]. After the initial assessment, (possible) problems should be discussed with the patient and/or informal caregiver to develop tailored aims for further assessment, treatment and/or follow-up. A specific advantage of an observation stay, is the opportunity for patient reassessment. For researchers, this unexplored territory can deliver dynamic predictors for vulnerability algorithms that possibly outperform classic geriatric screening tools [45, 47].

## Plan for treatment and follow-up

It is noteworthy that only one study reported using a type 1 observation unit structure (i.e. an EOU with a dedicated space for observation and clearly predetermined protocols to guide clinical care, as defined by Ross and colleagues [16]), which is considered superior to the three other types that are not protocol-based, lack a dedicated space or have neither [23]. Although one might say that protocol-driven EOUs can only admit older patients with a (working) diagnosis corresponding to a regular available protocol (e.g. low-risk chest pain protocol), it is also possible to develop specific, stand-alone geriatric protocols (e.g. frailty protocol, fragility fracture protocol). So, clinicians favoring protocol-driven observation care need to make a conceptual choice when initiating a geriatric approach: either add geriatric evaluation to existing protocols as a modular component or develop stand-alone geriatric protocols and possibly allow a patient to be observed according multiple protocols at once.

Since EOUs are pivotal points between primary, inpatient, outpatient, intermediate and residential care, it is important that different networks are available to smoothen care transitions (e.g. automated health data transfer). Obviously, proper arrangements with ambulance services are necessary, as well, to ensure that patients can leave the EOU as soon as possible.

Clinicians considering to “geriatricize” their EOU or start a geriatric-focused observation unit can use for this purpose the accreditation framework for geriatric emergency departments [51], the “Silver book” [7] or the McCusker framework [52]. As these documents offer a range of possibilities to enhance the care for older adults with emergency care needs, stepwise integration of quality improvement initiatives using properly selected implementation strategies seems recommended [53].

## Limitations and strengths

Following methodological limitations of this study need to be considered when interpreting the study results. First, possibly not all relevant studies were identified, as the search was limited to three databases and did not include grey literature. Theoretically, some papers which did not report having a geriatric focus in its emergency observation unit could have been improperly excluded. However, we estimate these odds are relatively small as geriatric emergency care initiatives are rather novel and emerging. Another restriction regarding retrieved articles could be due to the language skills of the research team (i.e. only studies in English, French and Dutch were considered for inclusion). Second, the last stage of the revised methodological framework for scoping reviews (i.e. consultation of stakeholders for study finding validation) was not performed [20, 21]. However, this stage was reported to be optional. Strengths of this study are the rigorous application of the essential stages in the methodological framework for scoping reviews, the systematic literature search and assessment of study quality.

## Conclusion

This scoping review documented that the structure and processes of EOUs with a geriatric focus are very heterogeneous and rarely cover all elements of CGA. Further research is necessary to determine how complex care principles of ‘observation medicine’ and ‘(C)GA’ can ideally be merged and successfully implemented in clinical care.

## Supplementary Information

**Additional file 1.**

## Data Availability

All data generated or analysed during this study are included in this published article and its supplementary files.

## Abbreviations

CGA: Comprehensive Geriatric Assessment
ED(s): Emergency Department(s)
e.g.: Example given
EOU(s): Emergency Observation Unit(s)
i.e.: Id est
LOS: Length Of Stay
MINORS: Methodological Index for Non-Randomized Studies
PRISMA: Preferred Items for Systematic Review and Meta-Analyses
TIDieR: Template for Intervention Description and Replication
UK: United Kingdom
USA: United States of America
