# Supplementary material for: Structure and processes of emergency observation units with a geriatric focus: a scoping review
Source: BMC Geriatr. 2021 Feb 1;21:95. doi: 10.1186/s12877-021-02029-9 (PMC7852183; doi:10.1186/s12877-021-02029-9)
Supplement: Supplementary file 1 — Additional file 1. [file 12877_2021_2029_MOESM1_ESM.docx]

**SUPPLEMENTARY TABLES**

- **SUPPLEMENTARY TABLE S1. Final search strings per database on March 5^th^, 2020**
- **SUPPLEMENTARY TABLE S2. Quality appraisal of included studies with Methodological Index for Non-Randomized Studies**
- **SUPPLEMENTARY TABLE S3. Design of emergency observation units with a geriatric focus**
- **SUPPLEMENTARY TABLE S4. Staffing of emergency observation units with a geriatric focus**
- **SUPPLEMENTARY TABLE S5. Admission policy of emergency observation units with a geriatric focus**

**SUPPLEMENTARY TABLE S1. Final search strings per database on March 5^th^, 2020**

| **MEDLINE**  **(n=2193)** | ((Emergency Medical Services[Mesh] OR Emergency Medical Service*[tiab] OR Emergency health service*[tiab] OR Emergency Treatment[Mesh] OR Emergency Treatment*[tiab] OR Emergencies[Mesh] OR "Emergencies"[tiab] OR "emergency"[tiab] OR Emergency Service, Hospital[Mesh] OR "Hospital Emergency Service"[tiab] OR Emergency Nursing[Mesh] OR "Emergency Nursing"[tiab] OR "emergency care"[tiab] OR emergency department*[tiab] OR emergency room*[tiab] OR emergency ward*[tiab] OR emergency unit*[tiab] OR "ED"[tiab] OR "EDs"[tiab] OR Acute Medical Unit*[tiab]) **AND** (aged[Mesh] OR "Aged"[tiab] OR "geriatric care"[tiab] OR "Gerontologic Care"[tiab] OR Geriatric Nursing[Mesh] OR "Geriatric Nursing"[tiab] OR "Gerontologic Nursing"[tiab] OR "elderly"[tiab] OR "elder"[tiab] OR "elders"[tiab] OR older patient*[tiab] OR older person*[tiab] OR "older people"[tiab] OR older adult*[tiab] OR "senior"[tiab] OR "seniors"[tiab] OR geriatric patient*[tiab] OR “geriatric”[tiab]) **AND** (Clinical Observation Units[Mesh] OR observation unit*[tiab] OR observation stay*[tiab] OR short stay hospital*[tiab] OR short stay unit*[tiab] OR short stay*[tiab] OR assessment unit*[tiab] OR frailty unit*[tiab] OR "observation medicine"[tiab] OR "observation status"[tiab] OR "Observation"[tiab])) |
| --- | --- |
| **EMBASE**  **(n=4143)** | ('emergency medical services':ti,ab,kw OR 'emergency health service'/exp OR 'emergency health service*':ti,ab,kw OR 'emergency treatment'/exp OR 'emergency treatment*':ti,ab,kw OR 'emergencies':ti,ab,kw OR 'emergency'/exp OR 'emergency':ti,ab,kw OR 'hospital emergency service'/exp OR 'hospital emergency service*':ti,ab,kw OR 'emergency nursing'/exp OR 'emergency nursing':ti,ab,kw OR 'emergency care'/exp OR 'emergency care':ti,ab,kw OR 'emergency department*':ti,ab,kw OR 'emergency room*':ti,ab,kw OR 'emergency ward'/exp OR 'emergency ward*':ti,ab,kw OR 'emergency unit*':ti,ab,kw OR 'ed':ti,ab,kw OR 'eds':ti,ab,kw OR 'acute medical unit*':ti,ab,kw) **AND** ('aged'/exp OR 'aged':ti,ab,kw OR 'geriatric care'/exp OR 'geriatric care':ti,ab,kw OR 'gerontologic care':ti,ab,kw OR 'geriatric nursing'/exp OR 'geriatric nursing':ti,ab,kw OR 'gerontologic nursing':ti,ab,kw OR 'elderly':ti,ab,kw OR 'elder':ti,ab,kw OR 'elders':ti,ab,kw OR 'older patient*':ti,ab,kw OR 'older person*':ti,ab,kw OR 'older people'/exp OR 'older people':ti,ab,kw OR 'older adult'/exp OR 'older adult*':ti,ab,kw OR 'senior':ti,ab,kw OR 'seniors':ti,ab,kw OR 'geriatric patient*':ti,ab,kw OR 'geriatric':ti,ab,kw) **AND** ('observation unit'/exp OR 'observation unit*':ti,ab,kw OR 'observation stay*':ti,ab,kw OR 'short stay hospital'/exp OR 'short stay hospital*':ti,ab,kw OR 'short stay unit'/exp OR 'short stay unit*':ti,ab,kw OR 'short stay*':ti,ab,kw OR 'assessment unit*':ti,ab,kw OR 'frailty unit*':ti,ab,kw OR 'observation medicine':ti,ab,kw OR 'observation status':ti,ab,kw OR 'Observation':ti,ab,kw) |
| **CINAHL**  **(n=802)** | MH "Emergency Medical Services+" OR TI "Emergency Medical Service*" OR AB "Emergency Medical Service*" OR TI "emergency health service*" OR AB "emergency health service*" OR MH "Emergency Treatment+" OR TI "Emergency (Treatment*" OR AB "Emergency Treatment*" OR MH "Emergencies+" OR TI "Emergencies" OR AB "Emergencies" OR TI "Emergency" OR AB "Emergency" OR MH "Emergency Service+" OR TI "Emergency Service*" OR AB "Emergency Service*" OR MH "Emergency Nursing+" OR TI "Emergency Nursing" OR AB "Emergency Nursing" OR MH "Emergency Care+" OR TI "Emergency Care" OR AB "Emergency care" OR TI "Emergency Department*" OR AB "Emergency Department*" OR TI "Emergency Room*" OR AB "Emergency Room*" OR TI "Emergency Ward*" OR AB "Emergency Ward*" OR TI "Emergency Unit*" OR AB "Emergency Unit*" OR TI "ED" OR AB "ED" OR TI "EDs" OR AB "EDs" OR TI "Acute Medical Unit*" OR AB "Acute Medical Unit*") **AND** (MH "Aged+" OR TI "Aged" OR AB "Aged" OR TI "Geriatric Care" OR AB "Geriatric Care" OR MH "Gerontologic Care" OR TI "Gerontologic Care" OR AB "Gerontologic Care" OR TI "Geriatric Nursing" OR AB "Geriatric Nursing" OR MH "Gerontologic Nursing+" OR TI "Gerontologic Nursing" OR AB "Gerontologic Nursing" OR TI "Elderly" OR AB "Elderly" OR TI "Elder" OR AB "Elder" OR TI "Elders" OR AB "Elders" OR TI "Older Patient*" OR AB "Older Patient*" OR TI "Older Person*" OR AB "Older Person*" OR TI "Older People" OR AB "Older People" OR TI "Older Adult*" OR AB "Older Adult*" OR TI "Senior" OR AB "Senior" OR TI "Seniors" OR AB "Seniors" OR TI "Geriatric Patient" OR AB "Geriatric Patient" OR TI "Geriatric" OR AB "Geriatric") **AND (**MH "Observation Units" OR TI "Observation Unit*" OR AB "Observation Unit*" OR TI "Observation Stay*" OR AB "Observation Stay*" OR TI "Short Stay Hospital*" OR AB "Short Stay Hospital*" OR TI "Short Stay Unit*" OR AB "Short Stay Unit*" OR TI "Short Stay*" OR AB "Short Stay*" OR TI "Assessment Unit*" OR AB "Assessment Unit*" OR TI "Frailty Unit*" OR AB "Frailty Unit*" OR TI "Observation Medecine" OR AB "Observation Medecine" OR TI "Observation Status" OR AB "Observation Status" OR TI "Observation" OR AB "Observation") |

**SUPPLEMENTARY TABLE S2. Quality appraisal of included studies with Methodological Index for Non-Randomized Studies**

|  | Anpalahan 2002  [36] | Bruun 2018  [32] | Chu 2007  [26] | Conroy 2014  [27] | Edmans 2013  [28] | Foo 2012  [38] | Khan 1997  [29] | Leung 2019  [39] | Misch 2014  [40] | Nielsen 2018  [33] | Ong 2012  [37] | Silvester 2012  [30] | Southerland 2018  [41] | Strøm 2017  [34] | Strøm 2018  [35] | Taylor 2016  [31] |
| --- | --- | --- | --- | --- | --- | --- | --- | --- | --- | --- | --- | --- | --- | --- | --- | --- |
| **A clearly stated aim** | 2 | 2 | 1 | 2 | 2 | 2 | 1 | 1 | 2 | 2 | 1 | 1 | 1 | 2 | 2 | 1 |
| **Inclusion of consecutive patients** | 1 | 2 | 2 | 2 | 1 | 1 | 2 | 1 | 2 | 1 | 1 | 2 | 1 | 1 | 1 | 2 |
| **Prospective data collection** | 1 | 2 | 2 | 1 | 2 | 2 | 1 | 1 | 2 | 2 | 1 | 2 | 1 | 2 | 2 | 1 |
| **Endpoints appropriate to the aim of the study** | 1 | 2 | 1 | 2 | 2 | 2 | 1 | 2 | 2 | 2 | 2 | 2 | 1 | 2 | 2 | 2 |
| **Unbiased assessment of the study endpoint** | 0 | 1 | 2 | 2 | 2 | 1 | 1 | 2 | 2 | 2 | 1 | 1 | 0 | 2 | 2 | 0 |
| **Follow-up period appropriate to study aim** | 2 | 2 | 2 | 2 | 2 | 2 | 1 | 2 | 2 | 2 | 2 | 2 | 2 | 2 | 2 | 2 |
| **Loss to follow up less than 5%** | 0 | 1 | 1 | 2 | 1 | 1 | 0 | 0 | 2 | 2 | 0 | 2 | 0 | 2 | 2 | 0 |
| **Prospective calculation of the study size** | 0 | 2 | 0 | 2 | 2 | 0 | 0 | 0 | 0 | 2 | 0 | 0 | 0 | 0 | 2 | 0 |
| **An adequate control group** | NA | 2 | NA | 1 | 2 | 1 | NA | 1 | NA | 1 | 1 | NA | NA | 1 | 2 | 0 |
| **Contemporary groups** | NA | 2 | NA | 1 | 2 | 1 | NA | 2 | NA | 2 | 0 | NA | NA | 2 | 2 | 1 |
| **Baseline equivalence of groups** | NA | 2 | NA | 1 | 1 | 2 | NA | 1 | NA | 1 | 1 | NA | NA | 1 | 1 | 0 |
| **Adequate statistical analyses** | NA | 2 | NA | 2 | 2 | 2 | NA | 1 | NA | 2 | 2 | NA | NA | 2 | 2 | 2 |

The twelve items were scored NA (not applicable), 0 (not reported), 1 (reported but inadequate) or 2 (reported and adequate).

**SUPPLEMENTARY TABLE S3. Design of emergency observation units with a geriatric focus**

| **Study** | **Location** | **Dedicated area** | **Protocol availability** | **Hybrid unit*** | **Capacity** | **Accommodation, equipment and supplies** |
| --- | --- | --- | --- | --- | --- | --- |
| Anpalahan 2002[36] | NR | Yes | NR | NR | NR | NR |
| Bruun 2018[32] | ED^1^ | Yes | NR | NR | NR | NR |
| Chu 2007[26] | HB | Yes | NR | NR | 32 beds | NR |
| Conroy 2014[27] | ED^2^ | Yes | NR | NR | 8-12 beds | NR |
| Edmans 2013[28] | NR | Yes | NR | NR | NR | NR |
| Foo 2012[38] | ED^2^ | Yes | NR | NR | 24 beds | NR |
| Khan 1997[29] | ED^2^ | Yes | NR | NR | 8 beds | NR |
| Leung 2019[39] | ED^1^ | Yes | Yes | NR | 6 beds | NR |
| Misch 2014[40] | ED^1^ | Yes | NR | Yes | NR | NR |
| Nielsen 2018[33] | ED^1^ | NR | NR | NR | NR | NR |
| Ong 2012[37] | HB | Yes | NR | NR | 13 beds | NR |
| Silvester 2012[30] | HB | Yes | NR | NR | NR | NR |
| Southerland 2018[41] | ED^1^ | Yes | Yes | NR | 20 beds | NR |
| Strøm 2017[34] | ED^2^ | Yes | NR | NR | 16 beds and 6 chairs | NR |
| Strøm 2018[35] | ED^2^ | Yes | NR | NR | 16 beds and 6 chairs | NR |
| Taylor 2016[31] | NR | Yes | NR | NR | 12 beds, but flexible | NR |

*Hybrid units: these units allow the dedicated space to be used by both observation patients and other patient populations (e.g. recovering elective procedure patients).; ED^1^ = within main ED; ED^2^ = immediately next to ED; HB = hospital-based; NR = not reported.

**SUPPLEMENTARY TABLE S4. Staffing of emergency observation units with a geriatric focus**

|  | **PHYSICIAN** | | | | **NURSES** | | | | **ALLIED HEALTH CARE PROFESSIONALS** | | | | | |
| --- | --- | --- | --- | --- | --- | --- | --- | --- | --- | --- | --- | --- | --- | --- |
|  | **G** | **CP** | **EDP/**  **AP** | **JMS** | **N** | **ANP/**  **APP** | **NCM/ PCC** | **MHL** | **SW** | **PT** | **DPC** | **OT** | **P** | **UN** |
| Anpalahan 2002[36] |  | X |  | X | X | X |  |  |  |  |  |  |  | X |
| Bruun 2018[32] |  |  | X |  | X |  |  |  |  | X |  | X |  |  |
| Chu 2007[26] |  | X | X |  | X |  |  |  | X | X | X | X |  |  |
| Conroy 2014[27] | X | X | X |  | X |  | X |  |  | X |  | X |  |  |
| Edmans 2013[28] | X | X | X |  | X |  |  |  |  | X |  | X |  |  |
| Foo 2012[38] |  |  | X |  | X | X |  |  | X | X |  |  |  |  |
| Khan 1997[29] |  | X | X |  |  |  |  |  | X | X |  | X |  |  |
| Leung 2019[39] | X |  | X |  | X |  | X |  |  | X |  | X |  |  |
| Misch 2014[40] |  |  | X |  | X |  |  |  |  |  |  |  |  |  |
| Nielsen 2018[33] |  |  | X |  | X |  |  |  |  | X |  | X |  |  |
| Ong 2012[37] |  |  |  | X | X |  |  |  | X | X |  | X |  |  |
| Silvester 2012[30] | X |  |  | X | X |  |  |  | X | X |  | X | X |  |
| Southerland 2018[41] | X | X | X |  | X | X | X |  |  | X |  |  | X |  |
| Strøm 2017[34] |  |  | X |  |  |  |  |  |  | X |  | X |  |  |
| Strøm 2018[35] |  | X | X | X | X |  |  |  |  | X |  | X |  |  |
| Taylor 2016[31] | X | X | X | X | X |  | X | X | X | X |  | X | X |  |

G = geriatrician; CP = consultant physician (e.g. acute medical consultant, internal medicine consultant), EDP = emergency department physician, AP= acute physician, JMS = junior medical staff; N = nurse; ANP = advanced nurse practitioner; APP = advanced practice provider; NCM = nurse case manager; PCC = primary care coordinators; MHL=mental health liaison nurse; SW = social worker; PT = physiotherapist; DPC = discharge planning coordinator; OT = occupational therapist; P = pharmacist;, UN = unspecified

**SUPPLEMENTARY TABLE S5. Admission policy of emergency observation units with a geriatric focus**

| **Study** | **Time cutoff** | **Admission procedure** | **Process variation** | **Observation unit population** |
| --- | --- | --- | --- | --- |
| Anpalahan 2002[36] | 48 hours | NR | NR | Medical patients who are generally (but not necessarily) old with multisystem diseases |
| Bruun 2018[32] | 48-72 hours | NR | NR | Common complaints include infection, thromboembolic disease, musculoskeletal disease, cardiovascular disease, but not obvious signs of stroke or myocardial infarction. |
| Chu 2007[26] | 72 hours | Closed | NR | Patients of all ages (over 16 years) who are likely to be investigated, managed and discharged within 72 hours. |
| Conroy 2014[27] | 24 hours | NR | No; daily geriatrician coverage | Older people who are likely to be discharged home within 24 hours |
| Edmans 2013[28] | up to 72 hours | NR | NR | Patients with medical crises (no age-related criteria) |
| Foo 2012[38] | 4-24 hours | NR | Yes: Monday till Saturday | Following conditions are accepted: allergy, appendicitis, asthma, blunt trauma, cellulitis, gastroenteritis, gout, heart failure, head injury, hypoglycaemia, pneumonia, pyelonephritis and seizure |
| Khan 1997[29] | up to 24 hours | Closed | Yes: Monday till Friday | Patients who appear to need a brief period of assessment or treatment. Diagnoses of patients who are frequently discharged home are: falls, injury, infection, constipation, collapse, stroke or TIA, social problems |
| Leung 2019[39] | 72 hours | NR | NR | Older community-dwelling patients who were premobid independent for activities of daily living, with acute deconditioning due to an acute illness, increasing fall risk and need for post-discharge community support service |
| Misch 2014[40] | 24 hours | NR | NR | NR |
| Nielsen 2018[33] | 48 hours | NR | Yes: Monday till Friday | NR |
| Ong 2012[37] | 36-48 hours | Open | NR | Sub-acute, undifferentiated patients with complex or multiple co-morbidities with functional impairment. Patients with low acuity triage score who require further assessment and investigations with potential for discharge within 48 hours. |
| Silvester 2012[30] | NR | NR | No; daily geriatrician coverage | NR |
| Southerland 2018[41] | 24 hours | Closed | Yes: Monday through Saturday | Fulfilling criteria of 1 out of 37 protocols, including a protocol for patients who do not easily fit into any defined protocol. Criteria for consultations were left up to the ED physician. |
| Strøm 2017[34] | 72 hours | Closed | NR | Patients in whom a short stay is realistic according to physician’s assessment in the ED. Patients are discharged if there is no treatment ongoing and no tests should be applied on fast-track basis. |
| Strøm 2018[35] | 72 hours | Closed | Unclear | Patients with no immediate life-threatening disease (e.g. minor medical ailments, deterioration of chronic diseases or diffuse symptoms), capable of walking from bed to bathroom without assistance. Patients dependent on extensive nursing care are excluded. |
| Taylor 2016[31] | NR. Mean LOS ≈ 24 hours | Open | Monday till Friday 8:30am-5:00pm | Patients identified on referral to medicine with at least one of following criteria: falls, delirium, dementia or care home/intermediate care residents |

NR = Not reported; LOS = length of stay.
